# Epidemic spreading in modular time-varying networks

**DOI:** 10.1038/s41598-018-20908-x

**Published:** 2018-02-05

**Authors:** Matthieu Nadini, Kaiyuan Sun, Enrico Ubaldi, Michele Starnini, Alessandro Rizzo, Nicola Perra

**Affiliations:** 1 Department of Mechanical and Aerospace Engineering, New York University Tandon School of Engineering, Brooklyn, NY 11201 USA; 2 Laboratory for the Modeling of Biological and Socio-technical Systems, Northeastern University, Boston, MA 02115 USA; 3 Institute for Scientific Interchange, ISI Foundation, Turin, Italy; 4 Departament de Física Fondamental, Universitat de Barcelona, Martí i Franquès 1, 08028 Barcelona, Spain; 5 Universitat de Barcelona Institute of Complex Systems (UBICS), Universitat de Barcelona, Barcelona, Spain; 6 Dipartimento di Elettronica e Telecomunicazioni, Politecnico di Torino, Corso Duca degli Abruzzi 24, 10129 Torino, Italy; 7 Centre for Business Networks Analysis, University of Greenwich, London, UK

## Abstract

We investigate the effects of modular and temporal connectivity patterns on epidemic spreading. To this end, we introduce and analytically characterise a model of time-varying networks with tunable modularity. Within this framework, we study the epidemic size of Susceptible-Infected-Recovered, SIR, models and the epidemic threshold of Susceptible-Infected-Susceptible, SIS, models. Interestingly, we find that while the presence of tightly connected clusters inhibits SIR processes, it speeds up SIS phenomena. In this case, we observe that modular structures induce a reduction of the threshold with respect to time-varying networks without communities. We confirm the theoretical results by means of extensive numerical simulations both on synthetic graphs as well as on a real modular and temporal network.

## Introduction

Network thinking has become a prominent and convenient paradigm to unveil the properties of complex systems. Such a paradigm has been rapidly enriched, giving rise to variants that account for inherent features of real complex systems inferred by the availability of large, often time-resolved datasets^1–4^. Three are the main features that have captured the attention of researchers in the area. The first is heterogeneity in the statistical distributions of key topological properties such as the number of connections per node (degree) and the intensity of interactions (weight)^3,4^. This property is one of the hallmarks of complexity and is linked to a range of non-trivial dynamics^3,5^. For example, heterogeneity in the connectivity patterns makes networks extremely fragile to the spreading of infectious diseases and malicious attacks^6,7^. The second feature regards the presence of modules and communities^8^. Available datasets have highlighted that real networks are organized in modules, or communities, whereby the density of links *within* the community is much greater than the density of links *between* communities. On the one hand, communities can be treated as fairly independent entities within a large network, like the behaviour of different organs within the same body. On the other hand, from time to time, phenomena originating in a community may involve a huge portion, if not all, of the network. This is for example the case of pandemics originating from local outbreaks^6,9–11^. Nevertheless, the role played communities is still ambivalent. For example, the presence of communities might slow down or speed up the propagation of a disease and facilitate the spreading of social norms^12–20^. Detecting communities in a real system is not a trivial task, due to their fuzzy, vanishing, and overlapping nature. Also, in most of the available datasets communities are not explicitly labeled, so the validation of community detection algorithms cannot often be rigorously carried out^8,21^. Finally, networks are characterised by non trivial temporal dynamics^22,23^. The propensity of nodes to initiate and to attract interactions per unit time is typically heterogeneously distributed^24,25^. The same applies to the duration and the time interval between connections^13,26^. Furthermore, the creation or renewal of interactions might be correlated, and the dynamics driving the temporal evolution of networks are function of the time-scale considered^27,28^. However, the large majority of studies on dynamical processes unfolding on networks have been conducted under the hypothesis of *time scale separation* which effectively neglects all such features. In particular, the evolution of the process and the evolution of network are considered to take place at well-distinct time scales. Within this paradigm two opposite limits have been considered. In the first case, the dynamical process is assumed to be much faster than the evolution of the network. This is the limit of quenched/static networks^29^. Here, networks are fully characterised from an adjacency matrix, *A*_*ij*_ whose entries are non-zero for all connected pairs *i* − *j*^3^. The second case instead, is the opposite limit where an annealed version of the network can be considered and averaging (mean-field) techniques can be applied^30–32^. Annealed networks are fully characterised by an average adjacency matrix ${\bar{A}}_{{k}_{i},{k}_{j}}$ describing the probability of connection for nodes of degree *k*_*i*_ and degree *k*_*j*_^33^. In our case instead, the time scale regulating the dynamical process and the evolution of the network are comparable, the time scale approximation is not valid. This is the regime of time-varying networks^23^. Interestingly, the temporal nature of interactions might inhibit or facilitate spreading processes evolving at comparable time-scales^11,24,34–46^. The effects introduced by communities and time-varying connectivity patterns on dynamical processes have been mostly scrutinised separately. However, as few recent works pointed out, the two attributes are connected and their interplay introduces non-trivial effects, such as segregated behaviours and formation of hierarchical structures^47^; or the intricate competition between topological and temporal correlations^48^. The presence of groups, think for example the interaction network of students in a school, introduces specific dynamics that deeply affect spreading processes. A thorough modelling and study of these phenomena may be useful to define prioritisation of interventions and containment strategies in epidemic spreading^49^.

Altogether, these observations call for a general modelling framework aimed at characterising both features and single out their effects on real networks. The model presented in this paper leverages on the paradigm of Activity Driven Networks (ADNs) to model realistic temporal networks where the node and link dynamics coevolve at comparable time scales^24,50^ and includes the modularity phenomena, whereby connection patterns can be set to preferentially occur within a given community, rather than outside the community toward the rest of the population. While the coevolution of node and link dynamics is inherently considered in ADNs by construction, modularity is here modelled by a single parameter that regulates the interplay between the link formation within and outside of the community. In the context of epidemic processes on time-varying networks, our model is first characterised analytically. Then it is used to study the behaviour of different contagion processes on synthetic networks, and on a large, time-resolved dataset of scientific collaborations. Results and methods are discussed in detail in the following sections.

## Results

Here, we study the effect of modularity (i.e., the presence of communities in the network) on time-varying networks. To this extent, we introduce a model of time-varying networks with tunable modularity, able to capture several features of real temporal graphs. We derive an analytical characterisation of the model, and we study the behaviour of the Susceptible-Infected-Recovered (SIR) and the Susceptible-Infected-Susceptible (SIS) epidemic processes unfolding on its fabric^51^. Remarkably, while the presence of tightly connected clusters inhibits SIR processes, it favours the spreading of SIS-like diseases lowering the epidemic threshold. Interestingly, similar results have been recently obtained in models of time-varying networks characterised by correlated topological features induced by reinforcement of specific ties^42^. We confirm the theoretical picture emerging from synthetic networks by means of extensive simulations on a real word dataset of scientific collaborations within the American Physical Society (APS). Our results contribute to characterise the mechanisms, and their interplay, behind the complex, and often contradictory, behaviour of dynamical processes unfolding on real networks.

### Modular activity driven networks

**Figure 1:**
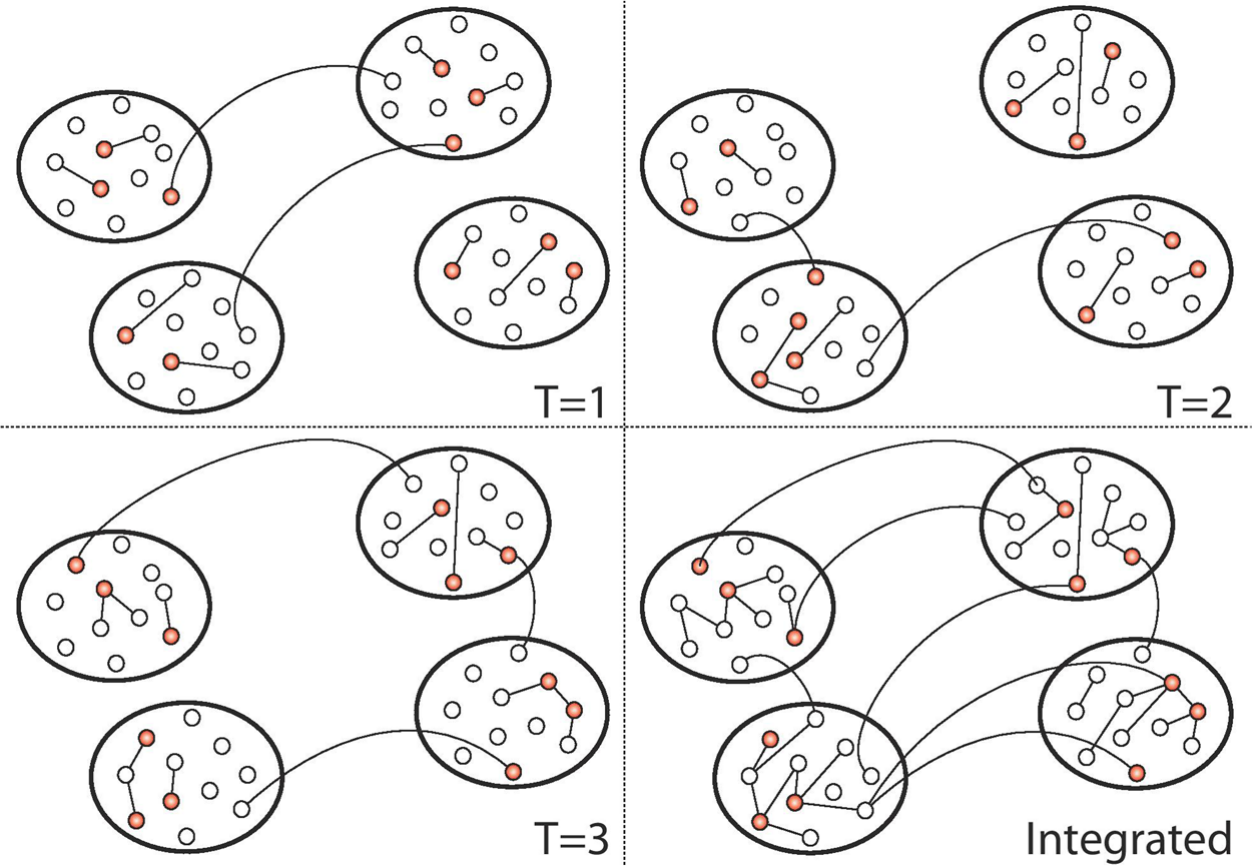
Schematic representation of the model. In red, we show active nodes. Straight lines and arcs describe links connecting nodes in the same or in different communities respectively. In the bottom right panel we show the integrated network obtained as the union of *G*_1_, *G*_2_, *G*_3_.

The system under investigation is composed by *N* nodes. Each node *i* is characterised by an activity rate *a*_*i*_, that describes its propensity to engage in social interactions with other nodes. To capture empirical observations performed in a wide set of systems ranging from R&D to online interactions networks^25,38,52,53^, we consider activity rates heterogeneously distributed, and extracted from a continuous functional form *F*(*a*) = *Ba*^−*ν*^, where *a* ∈ [*ε*, 1] and *ε* = 10^−3^ to avoid divergence in the distribution. Furthermore, each node is assigned to only one group/community. To take into account empirical evidences, the size of each community is extracted from a heavy-tailed distribution, i.e. *P*(*s*) = *Cs*^−*ω*^ with $s\in [{s}_{min},\sqrt{N}]$^8,54^. Therefore, we do not limit ourselves in studying a fixed number of modules^55^, whilst their number is driven from the model’s parameters. The assignment of nodes to communities is done as follow. A community of size *s* is extracted. The ID of *s* nodes is progressively assigned to the community. The processes is repeated until all *N* nodes are assigned to one community. Very rarely, all nodes can be perfectly assigned to the extracted community sizes. Indeed, in the last extraction we might have available only a fraction of the nodes necessary to fill the community. However, the average value of *s* is much smaller than the total number of nodes, thus the actual size of the last community can be only slightly smaller than the extracted value. The empirical distribution of community sizes in the network will then follow *P*(*s*). Given these settings, a generative network model is defined by the following steps (see Fig. 1).

- At each time *t*, the network, *G*_*t*_, starts with *N* disconnected nodes.
- With probability *a*_*i*_Δ*t* each vertex *i* is active and willing to create *m* connections.
- Each link being generated points with probability *μ* within the node’s community, and with probability 1 − *μ* to one of any other groups. In both cases, the target node *j* of the link is randomly selected in the target community.
- At the next time step *t* + Δ*t* all the edges in *G*_*t*_ are deleted.

All the interactions have a constant duration Δ*t*. In the model, neither self-loops nor multiple edges are allowed. In the following, without loss of generality, we fix Δ*t* = 1. Furthermore, we consider the case *m* = 1.

Given an heterogeneous distribution of activity, at each time step, the model generates a random, structureless network in which few nodes are active. The modular features of the network emerge integrating connections in time. Such time-integrated properties, at different time regimes, can be computed analytically. In the following, we will report the results for the evolution of the average number of connections of each node 〈*k*_*i*_(*t*)〉 (average degree) and the overall degree distribution *ρ*(*k*). The complete set of results is shown in the Supplementary Information (SI).

To solve the average degree’s dynamics, let us introduce the effective activity ${\tilde{a}}_{i}={a}_{i}+\langle a\rangle$ (where 〈*a*〉 is the average value of the activity distribution) and the mixing parameter *μ*′ = 1 − *μ*. We refer to the degree of node *i* at time *t* as *k*(*a*_*i*_, *s*, *t*), where *s* is the node’s community size. By defining an activity class as the group of nodes featuring similar activity values *a*, we set the average in-community degree 〈*k*_*c*_(*a*, *s*, *t*)〉 to be the average number of connections that nodes belonging to the activity class *a* and falling in communities of size *s* have toward nodes of their same community. The latter grows as

1 $$\langle {k}_{c}(a,s,t)\rangle =(s-\mathrm{1)}\,[1-\exp (-\frac{t}{\tau (a,s)})],$$

where *τ*(*a*, *s*) is the characteristic time that it takes for the degree *k*_*c*_(*a*, *s*, *t*) of nodes of activity *a* belonging to a community of size *s* to be ${k}_{c}(a,s,t)\sim (s-\mathrm{1)}$, being *s* − 1 the maximum value of the in-community degree (see the Supplementary Information for the evaluation of *τ*(*a*, *s*)).

Similarly, we can define the average out-community degree 〈*k*_*o*_(*a*, *t*)〉 as the number of connections that nodes of activity class *a* have outside of their communities at time *t*. We expect this quantity to be independent of the nodes’ community size *s* so that, for large networks, we can write:

2 $$\langle {k}_{o}(a,t)\rangle ={\mu }^{\text{'}}\tilde{a}t$$

The average total degree 〈*k*(*a*, *s*, *t*)〉 can be computed as the simple sum between the two previous equations, obtaining

$$\langle k(a,s,t)\rangle =\langle {k}_{c}(a,s,t)\rangle +\langle {k}_{o}(a,t)\rangle \simeq \{\begin{array}{lll}\tilde{a}t & t\ll \tau (a,s) & \quad \phantom{\rule{11em}{0ex}}(3{\rm{a}})\\ \mu ^{\prime} \tilde{a}t+(s-1) & t\sim \tau (a,s) & \quad \phantom{\rule{11em}{0ex}}(3{\rm{b}})\\ \mu ^{\prime} \tilde{a}t & t\gg \tau (a,s) & \quad \phantom{\rule{11em}{0ex}}(3{\rm{c}})\end{array}$$

**Figure 2:**
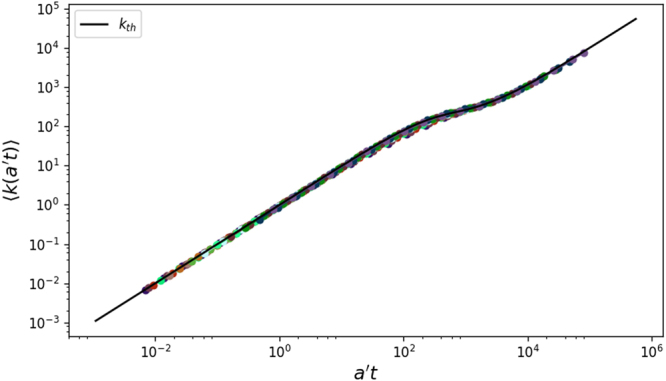
Time evolution of the average total degree, 〈*k*(*a*, *s*, *t*)〉, for different activity classes and compared with the theoretical function of Eq. 3a,b,c and, evaluated considering a community size equal to the average (i.e. *s* = 〈*s*〉). The rescaled time is $t\to \tilde{a}t$ and $\langle k(\tilde{a}t)\rangle$ is plotted. Parameters used are: *N* = 10^5^, *ω* = 2.1, *ν* = 2.1, *s*_*min*_ = 10, *μ* = 0.9 and *T* = 10^5^ evolution steps. Each point is an average of 10^2^ simulations.

Three regimes are readily identified: an initial growth in which both the in-community and the out-community degrees are growing linearly in time, followed by the slowing down of the in-community degree, which saturates to *s* − 1, and then a further linear regime driven only by the out-community degree growth. Figure 2 shows that the numerical simulations perfectly match with the theoretical formulas (see the SI for details).

Noticeably, the long time evolution of the node degree is linear in time and proportional to its activity class *a*, so that we find the asymptotic degree distribution of the system to feature the same functional form of *F*(*a*) ∝ *a*^−*ν*^, as found in non-modular activity driven networks^24,56^:

4 $$F(a)da\mathop{\longrightarrow }\limits^{k(a,t)\propto a\cdot t}\rho (k)dk\propto {k}^{-\nu }dk\mathrm{.}$$

**Figure 3:**
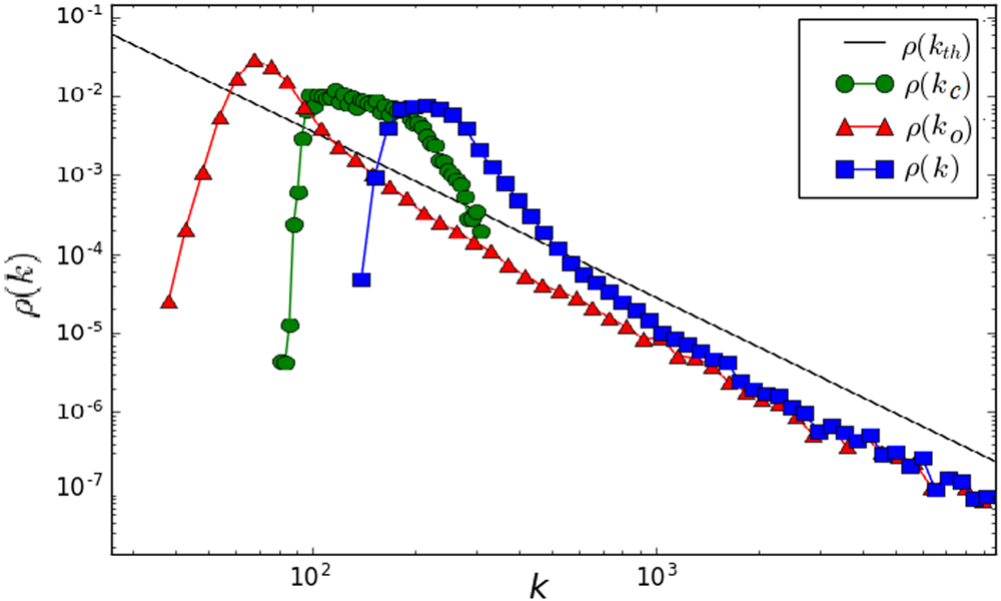
Plot of the three degree distributions and the theoretical prediction, given in Eq. 4. Parameters used are: *N* = 10^5^, *ω* = 2.1, *ν* = 2.1, *s*_*min*_ = 10, *μ* = 0.9 and *T* = 10^5^ evolution steps.

In Fig. 3, we integrate the network for *T* = 10^5^ and we plot the three degree distributions. As expected, the out-community *ρ*(*k*_*o*_) and the total *ρ*(*k*) degree distributions fall as power laws with exponent −*ν*. On the other hand, the in-community degree *ρ*(*k*_*c*_) saturates to the community size distribution *P*(*s*), as all the nodes reach their maximum in-community degree value (*s* − 1), being that the modules’ size is far smaller than the network size $({s}_{{\rm{\max }}}=\sqrt{N}\ll N)$. On the contrary, the out-community degree takes longer times to saturate to its maximum value $N-s\gg s$.

It is worth stressing that the results presented in this section apply to the networks obtained integrating links over time. A process unfolding on such networks, in general, will be affected by the time-aggregated features of the graph. The extent to which this is true, is function of the interplay between the time-scale describing its evolution, *τ*_*P*_, and the various *τ*(*a*, *s*). In the limit ${\tau }_{p}\ll \tau (a,s)$ the process would effectively evolve on the instantaneous, annealed networks that are characterised by a small average degree and modularity. In the opposite limit instead, the process would effectively unfold on static networks obtained integrating links over longer time characterised by high average degree and low modularity. Indeed, the average degree in this regime will be dominated by out-community links that make the connections between different communities increasingly stronger, thus increasingly destroying the identity of communities. In the limit ${\tau }_{p}\sim \tau (a,s)$ the process would effectively evolve on maximally modular networks (for a given set of parameters). Arguably, this is the most interesting regime that we will consider in the following.

### Epidemic processes on modular activity driven networks

Let us turn our attention on the dynamical properties of SIR and SIS processes (see the Methods section for a detailed definition of the two) unfolding on the proposed model. Although similar, the two processes are intrinsically different^33,57–59^. Indeed, SIR processes are always characterised by the so called disease-free equilibrium, provided *d*_*t*_*N* = 0. The illness eventually disappear, i.e., *I* = 0 for *t* → ∞. SIS models instead allow the existence of an endemic state where a finite and constant fraction of infected individuals permanently colonise the population, i.e., *I* > 0 for *t* → ∞. Here, we focus on a central concept of contagion phenomena: the epidemic threshold. This quantity defines the conditions necessary for the spreading of the illness. In annealed networks, the threshold is determined by the moments of the degree distribution *ρ*(*k*). In static graphs the expression is given by the principle eigenvalue of the adjacency matrix^57,60,61^. In time-varying networks instead, the threshold is determined by the interplay between the time-scales of the contagion and network evolution processes^24,41,45,50,62–69^. In the case of SIR models, we also consider another important quantity: the epidemic size *R*_∞_ which is defined as the final ratio of recovered nodes. This describes the fraction of nodes affected by the disease.

To develop a deeper understanding, let us derive the mean-field level dynamical equations describing the contagion process in modular activity driven networks. We define the activity block variables *S*_*a*,*s*_, *I*_*a*,*s*_, and *R*_*a*,*s*_ as the number of susceptible, infected and recovered individuals, respectively, in the class of activity *a* and community of size *s* at time *t* (to enhance readability, we omit to notate the dependence on time). This allows us to write the mean-field evolution of the number of infected individuals, for a SIR process, in each group of nodes with activity *a* as:

5 $$\begin{array}{rcl}{d}_{t}{I}_{a,s} & = & -\gamma {I}_{a,s}+\lambda {S}_{a,s}[\mu a\frac{{I}_{s}}{s}+\mathrm{(1}-\mu )a\frac{I}{N}]\\  &  & +\lambda \sum _{a^{\prime} }\,a^{\prime} [\mu {I}_{a^{\prime} ,s}\frac{{S}_{a,s}}{s}+\mathrm{(1}-\mu ){I}_{a^{\prime} ,s}\frac{{S}_{a,s}}{N}],\end{array}$$

where *I*_*s*_ and *I* are the number of infected in communities of size *s* and in the whole network, respectively. The first term in the r.h.s. accounts for the recovery of infected individuals. The other four terms account for the probability that a Susceptible node in a community of size *s* connects to an Infected node inside (first) or outside (second) its community acquiring the infection, and for the probability that an Infected node of class *a*′ connects to a Susceptible node inside (third) or outside (forth) a community of size *s*, contracting the disease. For simplicity, we consider that *N* − *s* ~ *N* and, at least initially, *I* − *I*_*s*_ ~ *I*. Summing over all the activities and community sizes, and considering only the first order terms in *a*, *I*_*a*,*s*_, *R*_*a*,*s*_ and their products, we obtain

6 $${d}_{t}I=-\gamma I+\lambda \langle a\rangle I+\lambda {\rm{\Theta }}+\lambda \mu \,\sum _{s}\,({\langle a\rangle }_{s}-\langle a\rangle ){I}_{s},$$

7 $$\begin{array}{rcl}{d}_{t}{\rm{\Theta }} & = & -\gamma {\rm{\Theta }}+\lambda \langle {a}^{2}\rangle I+\lambda \langle a\rangle {\rm{\Theta }}\\  &  & +\lambda \mu \,\sum _{s}\,[({\langle {a}^{2}\rangle }_{s}-\langle {a}^{2}\rangle ){I}_{s}+({\langle a\rangle }_{s}-\langle a\rangle {){\rm{\Theta }}}_{s}],\end{array}$$

where we defined ${\rm{\Theta }}={\sum }_{a}\,a{I}_{a}$, and ${{\rm{\Theta }}}_{s}={\sum }_{a}\,a{I}_{a,s}$. The term ${\langle {a}^{x}\rangle }_{s}={\sum }_{a}\,{N}_{a,s}{a}^{x}/s$ describes the moments of the activity distribution in any community of size *s*. The second, auxiliary, equation is obtained from the first by multiplying both sides by *a* and summing over all *s* and *a*. The epidemic threshold, in principle, can be derived evaluating the principle eigenvalue of the Jacobian matrix of the system of differential equations in *I* and Θ^24,41,45,50,69,70^. In general, a closed expression for the threshold does not exist. However, we can point out some interesting observations.

First of all, the terms associated to *R*_*a*,*s*_ vanish, implying that, at the first order, the thresholds of both SIR and SIS are equal^50^. Furthermore, the terms in *μ* weight a comparison between the moments of the activity distribution in the network with the corresponding quantities evaluated inside each community. If fluctuations of these terms are negligible, due for example to very large community sizes or to narrow distribution of activity, the equations become equivalent to the case *μ* = 0. In the limit *μ* → 0 the network has no modular structure, and the threshold, for both SIR and SIS, becomes $\beta /\gamma \ge \mathrm{2/}(1+\sqrt{\chi })$ as derived with different approaches in refs^24,41,45,71^. We defined *χ* = 〈*a*^2^〉/〈*a*〉^2^, where the moments are evaluated over the whole network. As expected, the spreading condition is determined by the interplay between the time-scale of the contagion process and the time-scale of the network. Furthermore, the threshold is significantly larger with respect to the case in which the disease would spread in static or annealed networks generated integrating connections over time^24^. Indeed, the concurrency of contacts in the two time scale separation regimes drastically facilitates the spreading of diseases^23,72^. It is important to notice how the threshold is not function of *m* even in the case of *m* > 1. This is due to the fact that we absorbed the contact rates in the definition of *β*. As detailed in the methods section, this is defined as the per capita rate of infection. By adopting such definition, we are able to estimate the spreading power of a disease independently of possible differences in contacts rates. The expression could be easily changed to explicitly account this aspect obtaining $\lambda /\gamma \ge \frac{1}{m\langle a\rangle \,(1+\sqrt{\chi })}$^24,46,73^. In the opposite limit *μ* → 1 networks are extremely modular, fluctuations become important and the symmetry between SIR and SIS breaks. In order to understand this limit, let us consider first a SIR process started from a single infected node in a community of size *s*. The large majority of connections are towards a small number of vertices in the same group. As soon as the disease start to spread within the community the number of infected and then recovered nodes grows, thus the probability of links *I* − *I* and *I* − *R* increases. Such connections cannot help the spreading of the disease. In fact they hamper the contagion process. In case instead of a network characterised by smaller values of modularity the growth of infected and recovered nodes has a much smaller effect. Indeed, the connectivity between communities would guarantee access to larger pool of susceptible nodes to sustain the spreading. From these simple observations we can expect that SIR processes are inhibited by highly modular connectivity patterns. Except for few exceptions in particular topologies^17^, this is the case on static and annealed graphs^12–17^. As we will show below, the same arguments hold also in time-varying connectivity patterns. On the other hand, in case of SIS processes, the repetition of contacts does not lead to such “pair annihilation”: contacts between infected nodes do not help the spreading of the disease, but they are only temporary (eventually, all infected nodes become susceptible again). Thus, we expect that modularity plays a different role in SIS dynamics. This is what has been found also in the case of one annealed network model^18^. Below we will show how this applies also in the case of time-varying networks. In order to numerically characterise SIR models, we study the epidemic size, *R*_∞_, as a function of *β*/*γ*. This quantity acts as the order parameter of a second-order phase transition^6^. For SIS processes instead, the order parameter is the final fraction of infected individuals, *I*_∞_^6^. The numerical estimation of this quantity is challenging, since it requires the precise determination of endemic states. For these reasons, we follow ref.^74^, measuring the life time of the disease, *L*, that acts as the susceptibility in phase transitions^74,75^. This quantity is defined as the average time it takes for the disease to either die out or reach a macroscopic fraction, *Y*, of the populations. Without loss of generality, we start our simulations by setting 1% of randomly selected nodes as initial infected seed. Other parameters are set as: *γ* = 0.01, *m* = 1, *ν* = 2.1, *ω* = 2.1, *N* = 10^5^ and *Y* = 0.5 (see SI for similar plots obtained fixing *ω* = 1.5).

**Figure 4:**
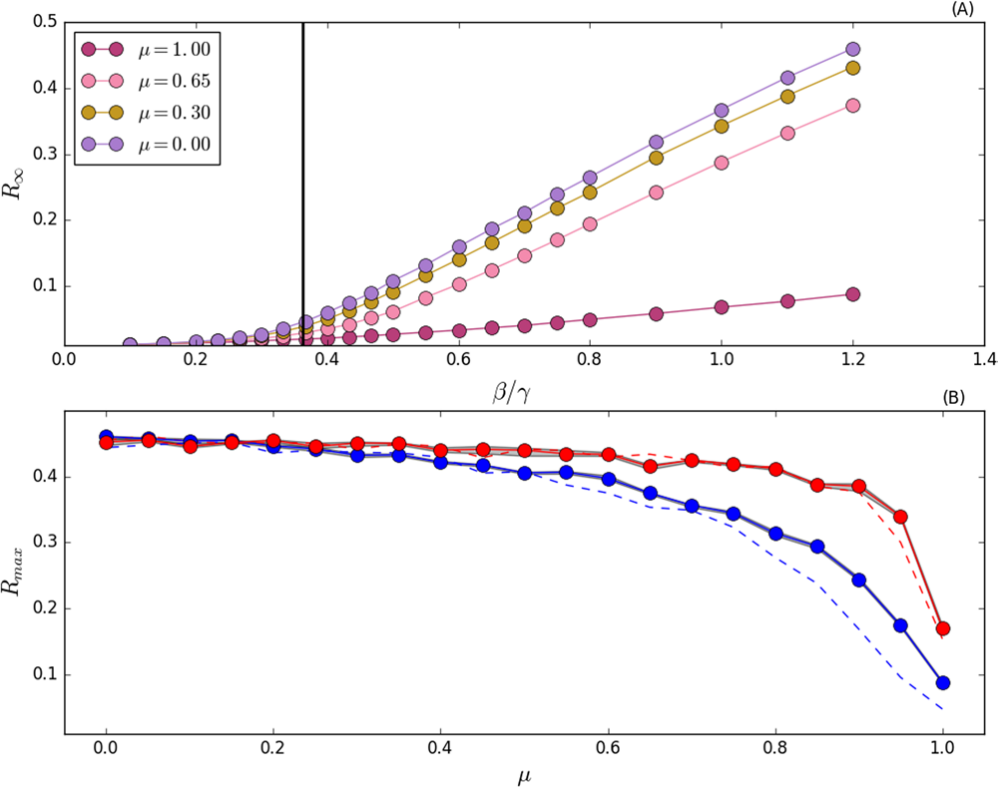
Panel (A) *R*_∞_ as a function of *β*/*γ*, for selected values of *μ* and *s*_min_ = 10. Vertical black line represents the theoretical value of the epidemic threshold for *μ* = 0 as derived in refs^24,71^. Panel (B) *R*_max_, i.e. the max value of *R*_∞_, as a function of *μ*. In red curves we set *s*_min_ = 100, in blue curves *s*_min_ = 10. In solid curves, we draw community sizes directly from the community size distribution *P*(*s*). In dashed curves, we fix the community sizes as equal to the average value of *P*(*s*) for all communities. The 95% confidence interval is in grey. Each point is an average of 10^2^ independent simulations.

**Figure 5:**
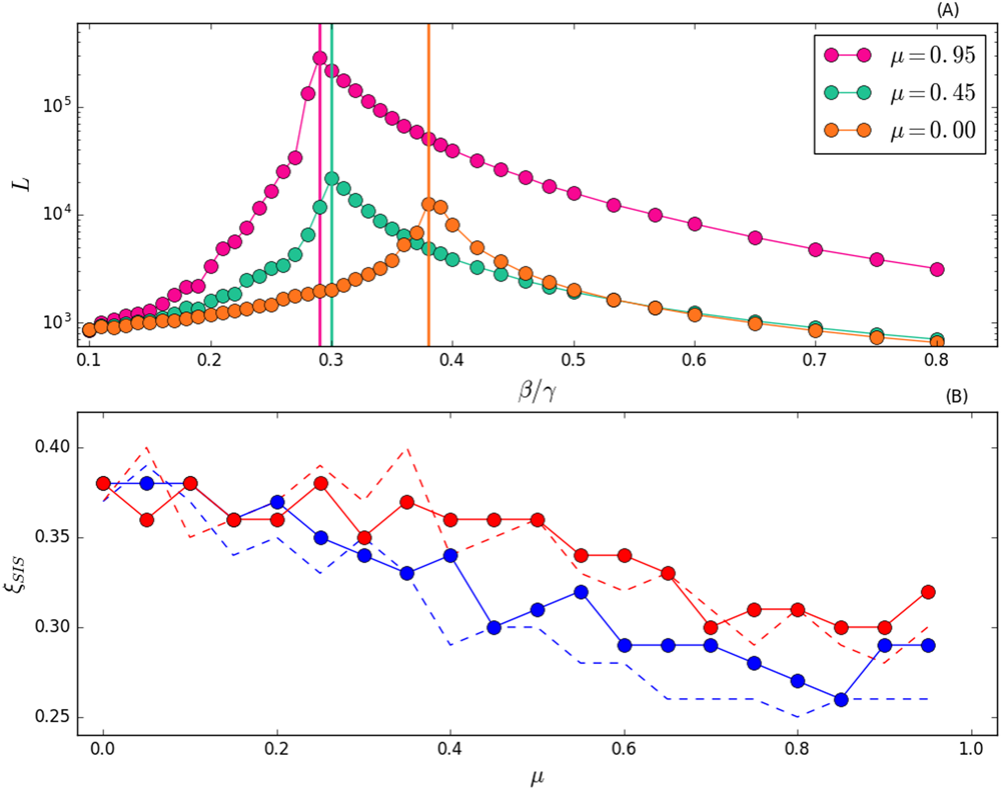
Panel (A) Lifetime of the disease *L* as a function of *β*/*γ*, for selected values of *μ* and when *s*_min_ = 10. Vertical lines are the epidemic threshold. Panel (B) Ratio *ξ*_SIS_ = *β*/*γ* in correspondence of *L*_max_, as a function of *μ*. In red curves we set *s*_min_ = 100, blue curves *s*_min_ = 10. Each point is an average of 10^2^ independent simulations. Note that we avoid to simulate *μ* = 1 because the criterion we follow for the estimation of the threshold does not hold for a network with many connected components.

Results obtained from SIR models are represented in Fig. 4A,B, whilst results from SIS models are shown in Fig. 5A,B. In Figs 4B and 5B we study different community structure, either by considering a constant community size (dashed curves) or by drawing community sizes directly from the community size distribution *P*(*s*) (solid curves). In general, red curves represents a network with bigger communities than the one represented with blue curves.

For SIR models, Fig. 4A tells us that, as expected, the higher *β*/*γ* the higher the epidemic size. The figure also confirm the intuitions about the threshold. Indeed, we observe a dependence on *μ*: the higher *μ* the higher the threshold. However, it is important to notice how such dependence is weak especially when compared with the SIS case (see below). Moreover, the higher the fraction of links created between pair of nodes sharing the same community (i.e. the higher *μ*), the lower the epidemic size. This second observation is confirmed studying different community structures, as done in Fig. 4B, in which we plot the maximum epidemic size (corresponding to the largest value of *β*/*μ* in our settings), *R*_max_, as a function of *μ*. In the limit *μ* → 0, we observe that the disease impact is the same: the networks behave as if no community structure was present. Instead, when *μ* → 1, the modular structure influences the spread of the disease. As mentioned before, repeating contacts within communities significantly narrows the chances of having new infected individuals. Indeed, in SIR models, once a node recovers, it cannot be infected again. Repeating contacts with nodes already recovered does not favour the spread of the disease. Overall, the main observations are four. (i) Increasing the modularity reduces the epidemic size. (ii) A network with, on average, larger modules is likely to yield a higher epidemic size. (iii) The larger the modules the weaker the dependence on *μ* of the epidemic size. (iv) In case of small modules, the distribution of community sizes seems to influence the spreading of the disease. In particular, a network organised in small groups of constant sizes leads to smaller epidemic size respect to a network in which the average community size is the same, but individual sizes are extracted from a power-law distribution.

For SIS models, the lower *μ*, the lower the life time *L* (see Fig. 5). Inter-community links speed up the disease spreading and an endemic state, i.e. *Y* = 0.5, is reached faster. Moreover, the higher *μ*, the lower the epidemic threshold. This last observation, which implies that increasing values of modularity favour the survival of the disease, is confirmed in Fig. 5B where we also test the effects of different community structures. In the limit *μ* → 0, there is no community structure and the curves converge to the same epidemic threshold. On the contrary, when *μ* → 1, the community structure becomes increasingly important and influences the spreading. Qualitatively, higher levels of modularity diminish the epidemic threshold. This is due to the repetition of the same contacts within a community which becomes increasingly more likely. Indeed, in SIS models, reinfection is allowed and nodes can become infected many times: communities act as a reservoir for the disease and favour the contagion process pushing the epidemic threshold to smaller values. Besides this last point, there are two main observations. (i) A network with larger modules is likely to have an higher epidemic threshold. (ii) In case of communities with smaller average sizes and high values of modularity, having the community size extracted from a power-law seems to slightly increase the threshold. Thus, the disease is able to spread more easily in modular networks with communities of similar or equal sizes. With the exception of one data point, this is observed for *μ* > 0.5 (see the dashed blue line in Fig. 5B).

### Real networks

**Figure 6:**
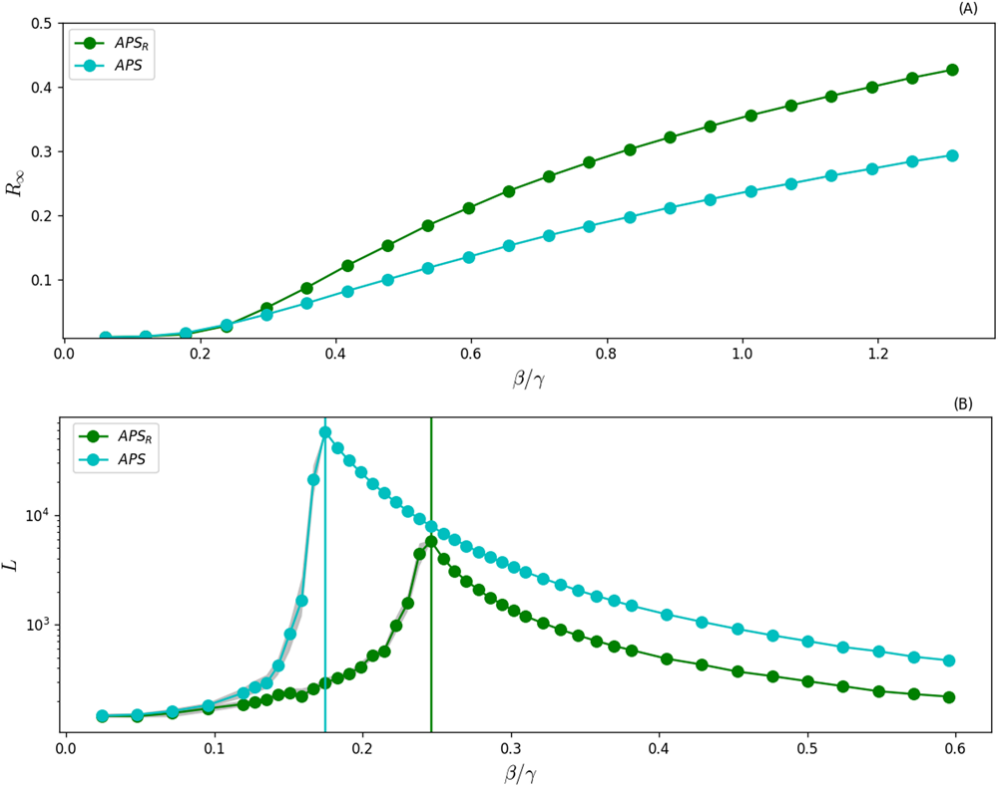
Panel (A) *R*_∞_ as a function of *β*/*γ* for SIR processes diffusing on APS (cyan circles) and on the randomized APS dataset (green circles). Panel (B) *L* as a function of *β*/*γ* for a SIS models evolving on the same two networks. Each point is the average of 10^2^ independent simulations started from 1% of random seeds. We fix *γ* = 0.05.

Although the modelling framework presented captures realistic activity and community size distributions of real networks, it neglects other important features such as burstiness^76–80^, and more complex temporal/structural correlations^81–85^. It is then crucial grounding the picture emerging from synthetic models with a real world system. To this extent, we consider a temporal and modular network about scientific collaborations in the American Physical Society (APS). We study 96940 scholars connected by 692667 links (see the Supplementary Information for more details)^86^. We focus on ten years of data (January 1997–December 2006) coarse-grained at a time resolution of one month. To single out the effects introduced by communities on contagion processes, we consider also a randomised version of the dataset. Here, the interactions at each time are shuffled, destroying the community structure, but the sequence of activation times for each node and the degree distribution at each time step are preserved^87^. In order to make sure that the randomisation process removes topological structures, we integrate the two networks over all time steps and we use OSLOM^88^ to find the communities. The modularity^89^ of the real APS network is *Q* = 0.6685, and of its randomised counterpart *Q* = 0.0937. As expected, the degree preserving randomisation reduces the modularity significantly. Using these two networks, we study the dynamical properties of SIR and SIS processes unfolding on their structure. In Fig. 6A,B we present the results. The modular properties of the real network do not influence the threshold of SIR models. Considering the weak dependence on the modularity observed in synthetic networks this results is not surprising. Even more, in this case the maximum value of modularity is defined by the data. We cannot increase it manually as done in our model. Nevertheless, the presence of communities reduces the impact of the disease, i.e. lowers the epidemic size. In the case of SIS processes instead, communities have a larger effect shifting the threshold to smaller values. These results qualitatively confirm what observed in synthetic systems.

## Discussion

Real networks are characterised by heterogeneous statistical distributions of crucial topological features; they are organised in modules/communities; they are subject to non trivial temporal dynamics^3–5,8,22,23^. It has long been acknowledged that such attributes have critical effects on contagion processes evolving on systems’ fabric^5^. In particular, the heterogeneity in the connectivity patterns makes static/annealed networks extremely fragile to the spreading of infectious diseases^6^. Moreover, the presence of communities in static/annealed graphs might either slow down or facilitate the propagation of a disease^12–18^. In particular, community structures are most likely to inhibit SIR-like processes^12–17^. However, in particular networks, such as Autonomous System Graphs, peculiar topological properties might have the opposite effect^17^. The study of SIS processes in the context of modular networks has received much less attention. However, at least in the case of one artificial network model, modularity has been found to help the spreading^18^. A note of caution is however important. Indeed, in this paper, modular networks are compared with random graphs of different degree distribution. Thus, it is hard to disentangle the real effect of modularity on SIS processes in this case. The temporal features of networks have been found to either facilitate or hamper the spreading of contagion processes. We refer the reader to ref.^90^ for a recent compendium of epidemic spreading on time-varying networks. The study of the effects of modularity on epidemic spreading unfolding on time-varying networks has been very limited. At the best of our knowledge only one paper so far tackled directly this issue^55^. In this work, an activity-driven network organised in two communities of equal size has been considered. In these settings, the threshold is not function of the modularity as each community is a good representation of the full network, thus the critical behaviour is the same independently of the number of connections between the two modules. Furthermore, the authors found a weak correlation between modularity and epidemic size. Starting from all these results, here we aimed to characterise the interplay between modularity and temporal dynamics of networks considering realistic community structures. To this end, we proposed a model of temporal networks with tunable modularity and heterogeneous activity distributions. We provided an analytical description of time-aggregated properties of such networks, and studied the impact of modular and temporal features on epidemic spreading processes. In synthetic networks, we found that modularity reduces the epidemic size and threshold in SIR models, slowing down the spreading process. The effect of modularity on the threshold is weak and appreciable only for large values of it. In SIS models, modularity reduces the epidemic threshold making the system more prone to disease spreading. The dependence on modularity is in this case much stronger. The repetition of the same contacts between nodes belonging to the same community acts as a reservoir for SIS-like diseases and allows the pathogen to reach an endemic state more easily. Our work is not exempt by caveats. The most important limitation, perhaps, is represented by the adoption of Poissonian activation dynamics, which has been shown to be unrealistic in empirical temporal networks which are characterised by bursty behaviours (heterogenous inter-event time distributions)^23^. Furthermore, not only links are dynamical entities being created and terminated, but also nodes may appear and disappear during the dynamics^23^. Our model of modular activity-driven networks does not capture all these aspects of real temporal networks. Thus, we confirmed the picture emerging in synthetic graphs by numerical simulations of SIR and SIS models on real network, characterised by both modular and temporal features. We selected a co-authorship network in which nodes describe authors and links between them capture scientific collaborations. Clearly, such network is not of direct epidemiological relevance. Unfortunately, datasets more suitable for the study of the behaviour of spreading processes on temporal networks, such as face-to-face interactions, are scarce and quite small in size. Thus, they are not the optimal choice to study how temporal and topological properties affect epidemic thresholds which are analytically defined in the limit of *N* → ∞. However, it is important to notice that social networks, in many different contexts, are characterised by the wide range of features missing in our simple model^3,23^. Thus, in the spirit of a qualitative comparison, studying the behaviour of contagion processes on co-authorship networks is a meaningful exercise. In conclusion, our findings show that on time-varying networks modularity can have opposite effects on different classes of spreading processes. Dynamical processes unfolding on real networks have been show to exhibit a rich and complex phenomenology, depending on the topological and temporal properties of the underlying substrate as well as on the characteristics of the process under investigation. Such complexity may be addressed by both the definition of proper generative models, that allows to control the desired features, and the study of the processes behaviour in real-world network. Our work is set within this framework, and it contributes to shed light on the impact of modular and temporal properties of real networks on epidemic spreading dynamics.

## Methods

### SIS and SIR models

In both processes nodes are divided in different classes according to their disease status. In SIR models nodes are either Susceptible (S), Infected (I) or Recovered (R). Susceptible nodes describe healthy individuals. Infected nodes contract the disease and are infectious. Recovered nodes are no longer infected and acquire complete immunity to the illness. The model is fully characterized by two transitions: $S+I\mathop{\to }\limits^{\beta }2I$ and $I\mathop{\to }\limits^{\gamma }R$. The first describes the infection propagation and *β* is the capita infection rate. This quantity is defined by the average contacts per node 〈*k*〉 and by the per contact probability of transmission *λ*, i.e. *β* = *λ*〈*k*〉. The second transition describes the recovery process. Infected individuals recover spontaneously and permanently with rate *γ*. In SIS models instead we have just Susceptible and Infected nodes. While the contagion process is equivalent to the SIR case, the recovery is different and described by the following transition: $I\mathop{\to }\limits^{\gamma }S$. Infected nodes spontaneously return in the susceptible compartment with rate *γ*.

## Electronic supplementary material

Supplementary Information

## References

[1] Barabási, A.-L. The network takeover. *Nat*. *Phys*. **8** (2012).

[2] Butts C (2009). Revisiting the foundations of network analysis. Science.

[3] Newman, M. *Networks*. *An Introduction* (Oxford University Press, 2010).

[4] Caldarelli, G. *Scale*-*Free Networks* (Oxford University Press, 2007).

[5] Barrat, A., Barthélemy, M. & Vespignani, A. *Dynamical processes on complex networks* (Cambridge, 2008).

[6] Vespignani A (2012). Modeling dynamical processes in complex socio-technical systems. Nat. Phys..

[7] Cohen R, Havlin S (2010). Complex Networks: Structure, Robustness and Function.

[8] Fortunato S (2010). Community detection in graphs. Phys. Reports.

[9] Colizza V, Vespignani A (2007). Invasion threshold in heterogeneous metapopulation networks. Phys. Rev. Lett..

[10] Apolloni A, Poletto C, Ramasco J, Jensen P, Colizza V (2014). Metapopulation epidemic models with heterogeneous mixing and travel behaviour. Theor. biology & medical modelling.

[11] Buscarino A, Fortuna L, Frasca M, Rizzo A (2014). Local and global epidemic outbreaks in populations moving in inhomogeneous environments. Phys. Rev. E.

[12] Onnela J-P (2007). Structure and tie strengths in mobile communication networks. Proc. Natl. Acad. Sci..

[13] Karsai M (2011). Small but slow world: How network topology and burstiness slow down spreading. Phys. Rev. E.

[14] Salathé M, Jones JH (2010). Dynamics and control of diseases in networks with community structure. PLoS computational biology.

[15] Huang W, Li C (2007). Epidemic spreading in scale-free networks with community structure. J. Stat. Mech. Theory Exp..

[16] Wu X, Liu Z (2008). How community structure influences epidemic spread in social networks. Phys. A: Stat. Mech. its Appl..

[17] Stegehuis, C., van der Hofstad, R. & van Leeuwaarden, J. S. Epidemic spreading on complex networks with community structures. *Sci*. *reports* **6** (2016). 10.1038/srep29748 PMC4954979 27440176

[18] Liu Z, Hu B (2005). Epidemic spreading in community networks. EPL (Europhysics Lett..

[19] Centola D (2010). The spread of behavior in an online social network experiment. Sci..

[20] Centola D, Baronchelli A (2015). The spontaneous emergence of conventions: An experimental study of cultural evolution. Proc. Natl. Acad. Sci..

[21] Kawadia V, Sreenivasan S (2012). Sequential detection of temporal communities by estrangement confinement. Sci. reports.

[22] Holme P, Saramäki J (2012). Temporal networks. Phys. Rep..

[23] Holme P (2015). Modern temporal network theory: a colloquium. The Eur. Phys. J. B.

[24] Perra N, Gonçalves B, Pastor-Satorras R, Vespignani A (2012). Activity driven modeling of dynamic networks. Sci. Reports.

[25] Alessandretti L, Sun K, Baronchelli A, Perra N (2017). Random walks on activity-driven networks with attractiveness. Phys. Rev. E.

[26] Barabasi A-L (2005). The origin of bursts and heavy tails in human dynamics. Nat..

[27] Barrat, A. & Cattuto, C. Face-to-face interactions. In *Social Phenomena*, 37–57 (Springer International Publishing, 2015).

[28] Sekara V, Stopczynski A, Lehmann S (2016). Fundamental structures of dynamic social networks. Proc. national academy sciences.

[29] Ódor G (2014). Slow, bursty dynamics as a consequence of quenched network topologies. Phys. Rev. E.

[30] Porfiri M, Stilwell DJ, Bollt EM, Skufca JD (2006). Random talk: random walk and synchronizability in a moving neighborhood network. Phys. D: Nonlinear Phenom..

[31] Frasca M, Buscarino A, Rizzo A, Fortuna L, Boccaletti S (2008). Synchronization of moving chaotic agents. Phys. Rev. Lett..

[32] Frasca M, Buscarino A, Rizzo A, Fortuna L (2012). Spatial pinning control. Phys. Rev. Lett..

[33] Ferreira SC, Castellano C, Pastor-Satorras R (2012). Epidemic thresholds of the susceptible-infected-susceptible model on networks: A comparison of numerical and theoretical results. Phys. Rev. E.

[34] Frasca M, Buscarino A, Rizzo A, Fortuna L, Boccaletti S (2006). Dynamical network model of infective mobile agents. Phys. Rev. E.

[35] Rocha LEC, Liljeros F, Holme P (2011). Simulated epidemics in an empirical spatiotemporal network of 50,185 sexual contacts. PLoS Comput. Biol.

[36] Isella L (2011). What’s in a crowd? Analysis of face-to-face behavioral networks. J. Theor. Biol.

[37] Miritello G, Moro E, Lara R (2011). Dynamical strength of social ties in information spreading. Phys. Rev. E.

[38] Karsai, M., Perra, N. & Vespignani, A. Time varying networks and the weakness of strong ties. *Sci*. *Reports* **4** (2014). 10.1038/srep04001 PMC3918922 24510159

[39] Scholtes, I. *et al*. Slow-down vs. speed-up of information diffusion in non-markovian temporal networks. *arXiv*:*1307*.*4030* (2013).

[40] Lambiotte R, Salnikov V, Rosvall M (2014). Effect of memory on the dynamics of random walks on networks. J. Complex Networks.

[41] Rizzo A, Frasca M, Porfiri M (2014). Effect of individual behavior on epidemic spreading in activity driven networks. Phys. Rev. E.

[42] Sun K, Baronchelli A, Perra N (2015). Contrasting effects of strong ties on sir and sis processes in temporal networks. The Eur. Phys. J. B.

[43] Rizzo A, Porfiri M (2016). Innovation diffusion on time-varying activity driven networks. EPJ B.

[44] Rizzo A, Pedalino B, Porfiri M (2016). A network model for ebola spreading. J. Theor. Biol..

[45] Zino L, Rizzo A, Porfiri M (2016). Continuous-time discrete-distribution theory for activity-driven networks. Phys. review letters.

[46] Speidel L, Klemm K, Eguiluz VM, Masuda N (2016). Temporal interactions facilitate endemicity in the susceptible-infected-susceptible epidemic model. New J. Phys..

[47] Liu M-X (2017). Social contagions on time-varying community networks. Phys. Rev. E.

[48] Artime, O., Ramasco, J. J. & San Miguel, M. Dynamics on networks: competition of temporal and topological correlations. *Sci*. *Reports* **7** (2017). 10.1038/srep41627 PMC5288700 28150708

[49] Stehle J (2011). High-resolution measurements of face-to-face contact patterns in a primary school. PLoS One.

[50] Liu S, Perra M, Karsai N, Vespignani A (2014). Controlling contagion processes in activity driven networks. Phys. Rev. Lett..

[51] Keeling, M. & Rohani, P. *Modeling Infectious Disease in Humans and Animals* (Princeton University Press, 2008).

[52] Tomasello, M. V., Perra, N., Tessone, C. J., Karsai, M. & Schweitzer, F. The role of endogenous and exogenous mechanisms in the formation of r&d networks. *Sci*. *reports* **4** (2014). 10.1038/srep05679 PMC4097357 25022561

[53] Ribeiro B, Perra N, Baronchelli A (2013). Quantifying the effect of temporal resolution on time-varying networks. Sci. Reports.

[54] Lancichinetti A, Fortunato S, Radicchi F (2008). New benchmark in community detection. Phys. Rev. E.

[55] Han D, Sun M, Li D (2015). Epidemic process on activity-driven modular networks. Phys. A: Stat. Mech. its Appl..

[56] Starnini M, Pastor-Satorras R (2013). Topological properties of a time-integrated activity-driven network. Phys. Rev. E.

[57] Castellano C, Pastor-Satorras R (2010). Thresholds for epidemic spreading in networks. Phys. Rev. Lett..

[58] Goltsev AV, Dorogovtsev SN, Oliveira JG, Mendes JFF (2012). Localization and spreading of diseases in complex networks. Phys. Rev. Lett..

[59] Sun, K., Baronchelli, A. & Perra, N. Epidemic spreading in non-markovian time-varying networks. *arxiv*:*1404*.*1006* (2014).

[60] Wang, Y., Chakrabarti, D., Wang, G. & Faloutsos, C. Epidemic spreading in real networks: An eigenvalue viewpoint. In *Proc 22nd Int*. *Symp*. *on Reliab*. *Distributed Syst*. 25–34 (2003).

[61] Durrett R (2010). Some features of the spread of epidemics and information on a random graph. Proc. Nat. Acad. Sci..

[62] Prakash B, Tong H, Valler M, Faloutsos C (2010). Virus propagation on time-varying networks: Theory and immunization algorithms. Mach. Learn. Knowl. Discov. Databases Lect. Notes Comput. Sci..

[63] Valdano E, Ferreri L, Poletto C, Colizza V (2015). Analytical computation of the epidemic threshold on temporal networks. Phys. Rev. X.

[64] Starnini M, Machens A, Cattuto C, Barrat A, Pastor-Satorras R (2013). Immunization strategies for epidemic processes in time-varying contact networks. J. Theor. Biol..

[65] Lee S, Rocha L, Liljeros F, Holme P (2012). Exploiting temporal network structures of human interaction to effectively immunize populations. PLoS One.

[66] Takaguchi T, Sato N, Yano K, Masuda N (2012). Importance of individual events in temporal networkss. New J. Phys..

[67] Tang, J., Mascolo, C., Musolesi, M. & Latora, V. Exploiting temporal complex network metrics in mobile malware containment. In *Proceedings of IEEE 12th International Symposium on a World of Wireless*, *Mobile and Multimedia Networks* (2011).

[68] Masuda, N. & Holme, P. Predicting and controlling infectious disease epidemics using temporal networks. *F1000Prime Reports* **5** (2013). 10.12703/P5-6 PMC3590785 23513178

[69] Pozzana, I., Sun, K. & Perra, N. Epidemic spreading on activity-driven networks with attractiveness. *arXiv preprint arXiv*:*1703*.*02482* (2017). 10.1103/PhysRevE.96.042310 PMC7217525 29347564

[70] Liu, S., Baronchelli, A. & Perra, N. Contagion dynamics in time-varying metapopulations networks. *Phy*. *Rev*. *E* **87** (2013).

[71] Starnini M, Pastor-Satorras R (2014). Temporal percolation in activity driven networks. Phys. Rev. E.

[72] Morris, M. *Sexually Transmitted Diseases*, (Holmes, K. K. *et al*. Eds) (McGraw-Hill, 2007).

[73] Onaga, T., Gleeson, J. & Masuda, N. Concurrency-induced transitions in epidemic dynamics on temporal networks. *Phys*. *Rev*. *Lett*. **108301** (2017). 10.1103/PhysRevLett.119.108301 28949155

[74] Boguña M, Castellano C, Pastor-Satorras R (2013). Nature of the epidemic threshold for the susceptible-infected-susceptible dynamics in networks. Phys. Rev. Lett..

[75] Aharony, A. & Stauffer, D. *Introduction to percolation theory* (Taylor & Francis, 2003).

[76] Ubaldi, E., Vezzani, A., Karsai, M., Perra, N. & Burioni, R. Burstiness and tie activation strategies in time-varying social networks. *Sci*. *Reports* **7** (2017). 10.1038/srep46225 PMC5390250 28406158

[77] Goh K-I, Barabási A-L (2008). Burstiness and memory in complex systems. EPL (Europhysics Lett..

[78] Moinet A, Starnini M, Pastor-Satorras R (2015). Burstiness and aging in social temporal networks. Phys. review letters.

[79] Lambiotte R, Tabourier L, Delvenne J-C (2013). Burstiness and spreading on temporal networks. The Eur. Phys. J. B.

[80] Karsai, M., Kaski, K., Barabási, A.-L. & Kertész, J. Universal features of correlated bursty behaviour. *Sci*. *reports* **2** (2012). 10.1038/srep00397 PMC3343322 22563526

[81] Peixoto, T. & Rosvall, M. Modelling sequences and temporal networks with dynamic community structures. *Nat*. *Commun*. **8** (2017). 10.1038/s41467-017-00148-9 PMC5605535 28928409

[82] Laurent G, Saramäki J, Karsai M (2015). From calls to communities: a model for time-varying social networks. The Eur. Phys. J. B.

[83] Pfitzner R, Scholtes I, Garas A, Tessone CJ, Schweitzer F (2013). Betweenness preference: Quantifying correlations in the topological dynamics of temporal networks. Phys. review letters.

[84] Vestergaard CL, Génois M, Barrat A (2014). How memory generates heterogeneous dynamics in temporal networks. Phys. Rev. E.

[85] Ubaldi, E. *et al*. Asymptotic theory of time-varying social networks with heterogeneous activity and tie allocation. *Sci*. *reports* **6** (2016). 10.1038/srep35724 PMC5075912 27774998

[86] Radicchi F, Fortunato S, Markines B, Vespignani A (2009). Diffusion of scientific credits and the ranking of scientists. Phys. Rev. E.

[87] Starnini M, Baronchelli A, Barrat A, Pastor-Satorras R (2012). Random walks on temporal networks. Phys. Rev. E.

[88] Lancichinetti, A., Radicchi, F., Ramasco, J. J. & Fortunato, S. Finding Statistically Significant Communities in Networks. *Plos One* (2011). 10.1371/journal.pone.0018961 PMC3084717 21559480

[89] Newman, M. E. J. & Girvan, M. Finding and evaluating community structure in networks. *Phys*. *Rev*. *E* **69** (2004). 10.1103/PhysRevE.69.026113 14995526

[90] Masuda, N. & Holme, P. Temporal network epidemiology (2017). 10.1098/rsif.2021.0019 PMC8169215 34062106

