# Supplementary material for: Epidemic spreading in modular time-varying networks
Source: Sci Rep. 2018 Feb 5;8:2352. doi: 10.1038/s41598-018-20908-x (PMC5799280; doi:10.1038/s41598-018-20908-x)
Supplement: Supplementary file 1 — Supplementary Information [file 41598_2018_20908_MOESM1_ESM.pdf]

Supplementary Information: epidemic spreading in modular time-varying networks

Matthieu Nadini[1,6], Kaiyuan Sun[2], Enrico Ubaldi[3], Michele Starnini[4,5], Alessandro Rizzo[6], and Nicola Perra[7,*]

[1]Department of Mechanical and Aerospace Engineering, New York University Tandon School of Engineering, Brooklyn NY 11201, USA
[2]Laboratory for the Modeling of Biological and Socio-technical Systems, Northeastern University, Boston MA 02115 USA
[3]Institute for Scientific Interchange, ISI Foundation, Turin, Italy
[4]Departament de Física Fondamental, Universitat de Barcelona, Martí i Franquès 1, 08028 Barcelona, Spain
[5]Universitat de Barcelona Institute of Complex Systems (UBICS), Universitat de Barcelona, Barcelona, Spain
[6]Dipartimento di Elettronica e Telecomunicazioni, Politecnico di Torino, Corso Duca degli Abruzzi 24, 10129 Torino, Italy
[7]Centre for Business Networks Analysis, University of Greenwich, London, UK
[*]n.perra@greenwich.ac.uk

ABSTRACT

In this Supplementary Information, we present in a more detailed and comprehensive way the definition of the model, its key properties and the results (both analytical and numerical) found. Furthermore, we show additional simulations studied in synthetic networks for SIR and SIS models. Finally, an explanation of the APS dataset is given.

1 The Model

The network is defined by means of the following parameters:

- the total number N of nodes in the network;
- the activity distribution parameters, i.e. the lower cut-off ε and the leading exponent ν, so that $F(a) \propto a^{-\nu}$ for $a \in [\varepsilon, 1]$;
- the lower cut-off $s_{\min}$, the upper limit $s_{\max}$ and the exponent ω describing the community size distribution, i.e. $P(s) \propto s^{-\omega}$ for $s \in [s_{\min}, s_{\max}]$;
- μ is the probability that, once active, a node will connect to a node inside the community, so that $\mu' = 1 - \mu$ is the probability to fire outside the community;

We initialize the network extracting N activity values from the activity distribution $F(a)$ and we then group the nodes in communities of size s drawn from a size-distribution $P(s)$. Once we initialized the network we let it evolve following the time-varying activity driven framework. At each time step t we start with N disconnected nodes. Each node gets active with probability $a_i dt$ at each time step dt and fire to a randomly chosen node inside (outside) its own community with probability μ (μ'). At time $t + dt$ we delete all the edges and repeat the above procedure. Each node i will then have a set of neighbors that have been contacted or have contacted the node during the network growth. The size of such a set is the integrated degree k of the node i. Of these k neighbors, k_c will be inside the i's community (in-community degree) and $k_o = k - k_c$ will be external to the community (out-community degree).

2 The Network growth

In the integrated network, each node i has a set of neighbors that have been contacted or have contacted the node during the network growth. The size of such a set is the integrated degree k. Of these k neighbors, k_c are inside the i's community (i.e. the in-community degree) and $k_o = k - k_c$ are external to the community (i.e. the out-community degree). Since the model is memoryless, the in-community degree k_c and the out-community degree k_o are decoupled and can, in fact, be treated separately. Even the activity potential a_i of each node can be "split" in two components: the in-community activity $\mu a_i = (1 - \mu')a_i$

and the complementary out-community $\mu' a_i$. Indeed, each node points, on average, a fraction μ of its own events toward the community, while the remaining μ' are directed outside the community itself. Each node experiences a mean field of activity, $\mu \langle a \rangle$, coming from the community (provided that the community is large enough) and a supplementary external field $\mu' \langle a \rangle$ coming from the rest of the network.

2.1 The network time scales

As a first insight, let us note that the in-degree time dependence can be easily approximated with a probabilistic consideration. Each node i of activity a_i, within a community of size s, has $s-1$ available edges. Now, for each time step of the dynamics, the edge e_{ij} is created with probability $\mu(a_i + a_j)/(s-1)$. Then, on average, each edge emanating from i is activated with probability $c(a_i,\mu)/(s-1) = \mu(a_i + \langle a \rangle)/(s-1)$, where $c(a_i,\mu)$ is the number of edges intra-community. The probability $P(a_i,\mu,s,t)$ for an edge pointing to i not to be activated after t time steps then reads:

$$P(a_i,\mu,s,t) = \left(1 - \frac{c(a_i,\mu)}{s-1}\right)^t . \tag{1}$$

Since the in-degree $k_c \in [0, s-1]$, we can write:

$$k_c(a_i,\mu,s,t) = (s-1)(1 - P(a_i,\mu,s,t)). \tag{2}$$

In the following, we always have the dependency on a_i, μ and t, so to simplify the notation we drop most of those parameters: $P(a_i,\mu,s,t) = P'(s)$, to avoid confusions with the community size distribution P(s). Also, $k_c(a_i,\mu,s) = k_c(s)$ and $c(a_i,\mu) = c$.

We note that Eq. 1 gives us an estimation of the characteristic time $\tau(s)$ that takes for a node of activity a_i to saturate the in-degree $k_c \to (s-1)$. Indeed, we can rewrite Eq. 1 as:

$$P'(s) = \exp\left[t \ln\left(1 - \frac{c}{s-1}\right)\right] \Rightarrow \tau(s) = -\left[\ln\left(1 - \frac{c}{s-1}\right)\right]^{-1} . \tag{3}$$

So, as expected, the saturation time (i.e. the typical time for k_c to be of the same order of s) increases as the activity μa_i decreases and/or the community size s grows.

Generalizing the above reasoning, the characteristic time for a community to have the majority of the nodes saturated is obtained by evaluating the probability $P_e(s)$ to create (on average) an edge e_{ij} in a community of size s in a single evolution step. In other words, it is the number of edges activated in one step divided by the total number of possible edges in the network:

$$P_e(s) = \frac{2s\mu \langle a \rangle}{s(s-1)} . \tag{4}$$

The probability for one edge not to be created after t time steps is then:

$$\bar{P}_e(s) = \left(1 - \frac{2\mu \langle a \rangle}{s-1}\right)^t \Rightarrow \bar{P}_e(s) = \exp\left[-\frac{t}{\tau_c(s)}\right] \Rightarrow \tau_c(s) = -\left[\ln\left(1 - \frac{2\mu \langle a \rangle}{s-1}\right)\right]^{-1} , \tag{5}$$

where $\tau_c(s)$ represents the typical time by which the majority of the nodes of a community has a degree $k_c \simeq s$.

Note that, in the evaluation of both $\tau(s)$ and $\tau_c(s)$, we did not take into account the difference between edges pointing to a more active node and the ones pointing to a less active one. Nevertheless, this is a simple estimation that, as we will show later, correctly catches the general behaviour of the in-degree k_c for any value of μ, a_i and s. Besides, when computing the key features of the evolving network, we are now able to distinguish the short time range $t \ll \tau_c(s)$ (in which $k_c \ll s$ for any activity value a_i) and the long time limit $t \gg \tau_c(s)$ (in which $k_c \sim s$ for any activity value a_i).

2.2 The Master Equation and the $P(a,k,t)$

We can now write down the Master Equation (ME) for the quantities $P_c(s,k_c)$ and $P_o(s,k_o)$, that is, the probability for a node of activity a_i belonging to a community of size s to have degree in (out) degree k_c (k_o) at time t. In general, $a_i \Delta t$ represents the probability the node i is active, where a_i is the activity rate of node i. Without loss of generality we will assume $\Delta t = 1$. To get the ME for the in-degree k_c distribution, we exploit also the time-dependence for a couple of passages:

$$\begin{aligned} P_c(s,k_c,t+1) = P_c(s,k_c,t)\left[1 - \mu \sum_j a_j\right] + P_c(k_c,t)\left[\frac{k_c}{s}\mu a_i + \frac{s-1}{s}\mu \sum_{j \nsim i} a_j + \mu \sum_{j \sim i} a_j\right] + \\ + P_c(s,k_c-1,t)\left[\frac{s-k_c}{s}\mu a_i + \frac{1}{s}\mu \sum_{j \nsim i} a_j\right] , \end{aligned} \tag{6}$$

where $\sum_{j\sim i}$ and $\sum_{j\nsim i}$ are respectively two contracted notations for the sum of all the first neighbors of node i and the sum of all nodes but the neighbors of node i. The first parenthesis indicates the probability that none of the nodes in the network fire. Third (sixth) term is the probability a node i, in the instantaneous network, is active and fires to a node where, in the integrated counterpart, there is already (isn't) a link. Four (seventh) term is the probability a node j not linked to i fires to any of the nodes but i (fires to i). Fifth factor is the probability a node j already linked to i fires.
After some algebra, ME can be written as:

$$P_c(s,k_c,t+1)-P_c(s,k_c,t)=-\left[P_c(s,k_c,t)-P_c(s,k_c-1,t)\right]\left(\frac{s-k_c}{s}\mu a_i+\frac{\mu}{s}\sum_{j\nsim i}a_j\right). \tag{7}$$

Now we pass to the continuum limit by considering $t\gg 1$ and $k\gg 1$. So the $l.h.s$ becomes simply the time derivative with respect to $P_c(s,k_c)$ and, to obtain a proper convergence of the results, we can expand the probability with respect to the incommunity degree up to second order.
In the regime $t\ll\tau(s)$, we can neglect $k_c\ll s$ and $1/s\sum_{j\nsim i}a_j\approx\langle a\rangle$.

$$\frac{\partial P_c(s,k_c)}{\partial t}=(\mu a+\mu\langle a\rangle)\left[\frac{\partial P_c(s,k_c)}{\partial k_c}-\frac{1}{2}\frac{\partial^2 P_c(s,k_c)}{\partial k_c^2}\right], \tag{8}$$

where we dropped the a_i index since we expect all the nodes of a given activity to behave in the same way. Now a is an activation rate and in the treatment we assume it takes small values to avoid that two nodes become active together.
The solution of Eq. 8 reads:

$$P_c(s,k_c)=C\exp\left[-\frac{(k_c-\mu(a+\langle a\rangle)t)^2}{2\mu(a+\langle a\rangle)t}\right], \tag{9}$$

where C is a normalization constant.
By following the same procedure, we recover the same results of Eq. 9 for the out-community degree $k_o(a,t)$, by substituting $\mu\to\mu'$ and $k_c\to k_o$:

$$P_o(s,k_o)=C\exp\left[-\frac{(k_o-\mu'(a+\langle a\rangle)t)^2}{2\mu'(a+\langle a\rangle)t}\right] \tag{10}$$

Since $N\gg s_{max}$, the out-degree $k_o\ll N$ for any time t of the process, thus we assume that Eq. 10 is valid for all the time scales analyzed. Also note that, as expected, the net effect of the mixing parameter μ' is just a time rescaling of the out-community and in-community activity, respectively.
Then, in the $t\sim\tau_c(s)$ time range is not possible to find an analytic formula, however simulations will be run to provide, at least, a qualitative behavior. In the $t\gg\tau_c(s)$ time limit the $P_c(s,k_c)$ converges to the $\delta(k_c-(s-1))$ distribution. In fact, all the nodes will have all their edges activated and the $P_c(s,k_c)$ time derivative goes to zero.

Let us now resume the results found in this section:

$$P_c(s,k_c)\propto\begin{cases}\exp\left[-\frac{(k_c-\mu(a+\langle a\rangle)t)^2}{2\mu(a+\langle a\rangle)t}\right] & \text{for } t\ll\tau_c(s) \quad (11a)\\ \delta(k_c-(s-1)) & \text{for } t\gg\tau_c(s) \quad (11b)\end{cases}$$

$$P_o(s,k_o)\propto\exp\left[-\frac{(k_o-\mu'(a+\langle a\rangle)t)^2}{2\mu'(a+\langle a\rangle)t}\right] \quad \text{for } \forall t \tag{12}$$

where we now distinguish between the in-community degree distribution $P_c(s,k_c)$ and the out-community degree distribution $P_o(s,k_o)$. The latter however, is independent on the community size and we can then define $P_o(s,k_o)=P_o(k_o)$.
So far we treated the two probability functions separately when, in fact, k_c and k_o are bound by the relation $k=k_c+k_o$. The total degree distribution $P(s,k)$ will then be determined by the convolution of both the $P_c(s,k_c)$ and $P_o(s,k-k_c)$:

$$P(s,k)=\int_0^k dk_c P_c(s,k_c)P_o(k-k_c) \tag{13}$$

where we integrate over all the possible arrangements of the k_c edges.
In the $t \ll \tau(s)$ limit, by substituting Eq. 11a and 12 in Eq. 13, we sum the two exponents getting:

$$P(s,k) = C\int_0^k dk_c \exp\left[-\frac{(k_c-\mu(a+\langle a\rangle)t)^2}{2\mu(a+\langle a\rangle)t} - \frac{(k-k_c-\mu'(a+\langle a\rangle)t)^2}{2\mu'(a+\langle a\rangle)t}\right] \tag{14}$$

where C is, again, a normalization constant.
By combining the two terms and after some algebra we get:

$$P(s,k) = C\int_0^k dk_c \exp\left[-\frac{(k_c-\mu k)^2}{2\mu\mu'(a+\langle a\rangle)t} - \frac{k^2-2k(a+\langle a\rangle)t-(a+\langle a\rangle)^2t^2}{2(a+\langle a\rangle)t}\right]. \tag{15}$$

The integration over k_c gives:

$$P(s,k) = C\left[\mathrm{Erf}\left(\frac{\mu' k}{\sqrt{2\mu\mu'(a+\langle a\rangle)t}}\right) - \mathrm{Erf}\left(\frac{\mu k}{\sqrt{2\mu\mu'(a+\langle a\rangle)t}}\right)\right]\exp\left[-\frac{k^2-2k(a+\langle a\rangle)t-(a+\langle a\rangle)^2t^2}{2(a+\langle a\rangle)t}\right] \tag{16}$$

where $\mathrm{Erf}(x)$ is the error function evaluated at x.
In the small time limit, if we want to evaluate the $P(k) = \int_{s_{\min}}^{s_{\max}} dsP(s)P(s,k)$, we have to consider $t \ll \min_s(\tau_c(s))$. In this way, for each community we can use Eq. 16 as the true value of the $P(s,k)$. The integration over the different community size s is then straightforward since the terms are independent on it, giving $P(k) = P(s,k)$. Note that this result holds for any value of μ, N and a.
The computation of $P(k)$ in the large time limit (i.e. $t \gg \langle\tau_c(s)\rangle$) is more complicated and we have to assume that $k_c = s-1$ for each node in a community of size s, otherwise Eq. 11b put everything equal to zero. The $P_o(s,k_o)$ will still be approximated by Eq. 12. The integral now reads:

$$P(k) = \int_{s_{\min}}^{s_{\max}} dsP(s)P(s,k) = C\int_{s_{\min}}^{s_{\max}} dsP(s)\exp\left[-\frac{(k-(s-1)-\mu'(a+\langle a\rangle)t)^2}{2\mu'(a+\langle a\rangle)t}\right], \tag{17}$$

The exponential can be written as:

$$\exp\left[-\frac{(k-\mu'(a+\langle a\rangle)t)^2+(s-1)[(s-1)-2(k-\mu'(a+\langle a\rangle)t))]}{2\mu'(a+\langle a\rangle)t}\right], \tag{18}$$

where the rise of new terms proportional to s^2 and sk makes it difficult to perform the integral.

We can, however, give a solution for the simple case $P(s) = \delta(s-\bar{s})$, when all the communities have equal size. First of all, we have, for large times, $P_c(s,k_c) = \delta(k_c-(s-1))$. Then:

$$P(s,k) = \begin{cases} P_o(k-(s-1)) & \text{for } k \geq s-1 \quad (19a)\\ 0 & \text{for } k < s-1. \quad (19b)\end{cases}$$

When $k < s-1$, for sure $k_c < s-1$ and the delta put everything equal to zero. In the other case $k_c = s-1$ with a certain probability $P_o(k-(s-1))$. Finally, equation 17 can be written as:

$$P(k) = \begin{cases} P_o(k-(\bar{s}-1)) & \text{for } k \geq \bar{s}-1 \quad (20a)\\ 0 & \text{for } k < \bar{s}-1 \quad (20b)\end{cases}$$

2.3 The average degree $\langle k(a,s,t)\rangle$

We can provide a simple expression for the nodes average degree belonging in different classes. As we already showed in Eq. 2, $\langle k_c(a,s,t)\rangle$ grows as:

$$\begin{aligned}\langle k_c(a,s,t)\rangle &= (s-1)\left(1-\exp\left(-\frac{t}{\tau(s)}\right)\right) = \\ &= (s-1)\left(1-P'(s)\right) = \\ &= (s-1)\left[1-\left(1-\frac{c}{s-1}\right)^t\right],\end{aligned} \tag{21}$$

and the $\langle k_o \rangle$ grows as the mean value of the distribution given in equation 12, and turns out to be independent on s.

$$\langle k_o(a,t) \rangle = \mu'(a + \langle a \rangle)t \tag{22}$$

The average total degree $k(a,s,t)$ for nodes of activity a belonging to communities of size s depends on the time scale we analyse the problem. For small times (i.e. $t \ll \langle \tau(s) \rangle_s$):

$$\begin{aligned}\langle k(a,s,t) \rangle &= \langle k_c(a,s,t) \rangle + \langle k_o(a,t) \rangle \approx \\ &\approx (s-1)\left(1 - 1 + \frac{\mu(a+\langle a \rangle)t}{s-1}\right) + (1-\mu)(a + \langle a \rangle)t = \\ &= (a + \langle a \rangle)t\end{aligned} \tag{23}$$

As time grows toward the regime of times comparable to the average (i.e. $t \sim \langle \tau(a,s,t) \rangle_s$), we cannot approximate the in-community degree anymore but we use directly equation 2:

$$\langle k(a,s,t) \rangle = (s-1)(1 - P'(s)) + \langle k_o(a,t) \rangle \,. \tag{24}$$

Then, the regime of large times (i.e. $t \gg \langle \tau(s) \rangle_s$) is:

$$\langle k(a,s,t) \rangle \approx s - 1 + \mu'(a + \langle a \rangle)t \approx \mu'(a + \langle a \rangle)t \tag{25}$$

The above equations then predict that the average degree has a linear growth proportional to $a + \langle a \rangle$ for short time limit (equation 23), then a transition for $t \sim \langle \tau(s) \rangle_s$ is followed by a second linear growth, valid for large times, proportional to $\mu'(a + \langle a \rangle)$. These regimes correspond to the initial growth, in which both the in-community and the out-community degrees are growing linearly in time, followed by the slowing down of the in-community degree which is saturating to $s-1$. Finally, the third regime is again linear and it is driven by the $\mu'(a + \langle a \rangle)$ coefficient: it means meaning that only the out-community degree is growing.

2.4 The degree distribution $\rho(k_{\text{th}})$

Now that we have the expression of the average degree, it is straightforward to write the degree distribution. At all the time scales we found $\langle k_{\text{th}} \rangle \propto Ct$, where C is a time-independent coefficient. Then, $k_{\text{th}} \propto at$, and it results in an equal increment in the activity and in the degree values ($da = dk_{\text{th}}$). If we use the change of variable rule, we obtain:

$$F(a)da = \rho(k_{\text{th}})dk_{\text{th}} \Rightarrow a^{-\nu}da = k_{\text{th}}^{-x}dk_{\text{th}} \Rightarrow x = \nu, \tag{26}$$

i.e., the degree distribution has the same exponent ν as the activity distribution function.

3 Comparison with numerical simulations

To check the analytical predictions of Section 2 we performed numerical simulation. In particular we realized 100 representations of a network featuring:

- $N = 10^5$ nodes with modularity $\mu = 0.9$ evolving for 10^5 evolution steps;
- activity potential distributed following the $F(a) \propto a^{-\nu}$ with $\nu = 2.1$ and $a \in [10^{-3}, 1]$ interval;
- power-law distributed community sizes $P(s) \propto s^{-\omega}$ with $\omega = 2.1$ and $s \in [10, \sqrt{N}]$.

In order to analyze the collective behavior of the nodes we group them by their activity and community size, thus defining b classes of nodes. We average over the representations of the network and for each class of nodes b we evaluate:

- $P_c(s,k)$, $P_o(k)$, $P(s,k)$ for $t \ll \tau_c(s)$ and $t \gg \tau_c(s)$;
- the average degree $\langle k_c(s) \rangle$, $\langle k_o \rangle$ and $\langle k(s) \rangle$;
- the degree distribution $\rho(k_{\text{th}})$.

In the main discussion, we already showed that some of the above measures have a great agreement with analytical predictions. To complete the discussion, we add below Fig. 1 which proves that also the in-community probability and the in-community degree perfectly matches our expectation. In panel B) we display $P_c(s,k)$ for $t \sim \tau_c$ even if it was impossible to obtain an exact result, the comparison demonstrates that the in-community probability starts to deviate from a Gaussian distribution.

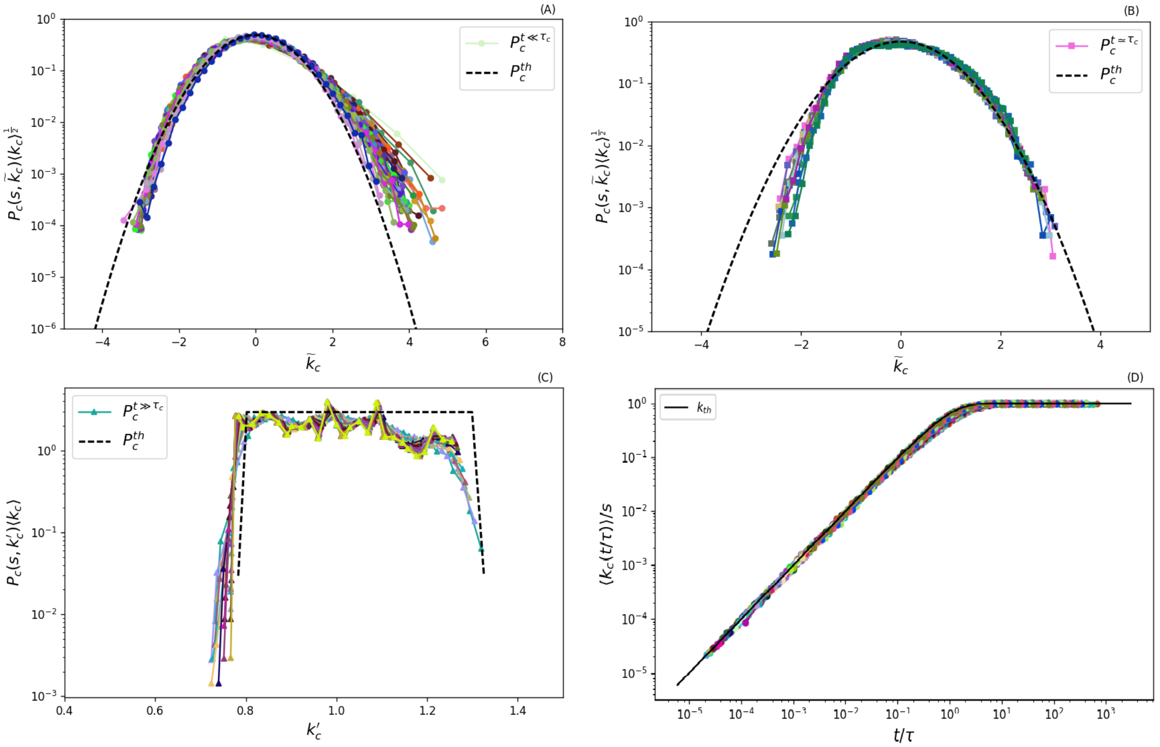

Figure 1. Panels A-C) rescaled $P_c(k_c)$ probability distribution as found for a selected node class at different times (legends). Functions in panels A-B) are rescaled accordingly to the theoretical distribution given in Eq. 11a, i.e., by sending $k_c \to \tilde{k_c} = (k_c - \langle k_c \rangle)/\langle k_c \rangle^{1/2}$ and plotting $P_c(\tilde{k_c})\langle k_c \rangle^{1/2}$. Panel C) is rescaled accordingly to the theoretical distribution given in Eq. 11b, i.e., by sending $k_c \to k_c' = k_c/\langle k_c \rangle$ and plotting $P_c(k_c')\langle k_c \rangle$. In panel D) we plot $\langle k_c \rangle$ for nodes featuring different activity potential and belonging to communities of different size. The data are rescaled sending the time $t \to= t/\tau$ and then plotting $\langle k_c(t/\tau) \rangle/s$. The analytical prediction of Eq. 2 computed for a node of activity $a = \langle a \rangle$ and belonging to a community of size $s = \langle s \rangle$. Each point is an average over 10^2 independent simulations, parameters used are: $N = 10^5$, $\omega = 2.1$, $\nu = 2.1$, $m = 1$, $s_{min} = 10$ and $\mu = 0.9$

4 SIR and SIS processes on modular activity driven networks

We present together all the results about SIR and SIS models obtained in synthetic networks. We start our simulations by setting 1% of randomly selected nodes as initial infected seeds, the other parameters are fixed as $\gamma = 0.01$, $m = 3$, $\nu = 2.1$, $N = 10^5$ and $Y = 0.5$. Panels A) and C) set the exponent of the distribution of community sizes $\omega = 1.5$, while panels B) and D) have $\omega = 2.1$. The qualitative picture is unchanged due to selecting a different value of ω. The modular structure becomes irrelevant for $\mu \rightarrow 0$, whilst it significantly modifies the spread of the disease when $\mu \gg 0$. Modularity slows down contagion processes in SIR models, it favors the disease outbreak in SIS models. Moreover, quantitatively, in SIR models the presence of larger communities lead to higher epidemic size, in SIS models it lower the epidemic threshold. Moreover, in SIS models and when $\mu \rightarrow 1$, the epidemic threshold is likely to increase due to major limitations in reaching an endemic size. This phenomenon is particularly visible in Panel C), solid blue curve.

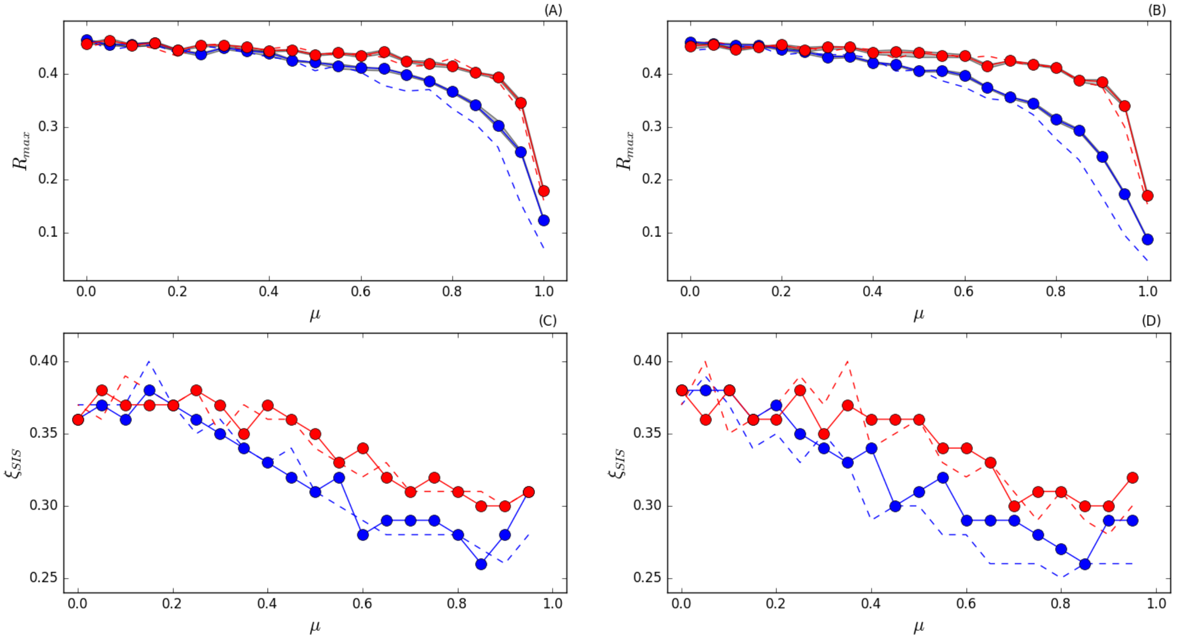

Figure 2. Panels A-B) take R_{max} of each R_∞ curve and plot it as a function of μ. Panels C-D) ξ_{SIS}, that is β/γ in correspondence of L_{max}, and plot it as a function of the modularity. In red curves we set $s_{min} = 100$, in blue curves $s_{min} = 10$. Solid curves are obtained by drawing community sizes from a power law distribution, 95% confidence interval is in gray. Dashed curves have a constant community size equal to the average value of the power law. Each point is an average of 10^2 independent simulations.

5 Real network: APS dataset

In the data each author of an article is described as a node. An undirected link between two different authors is drawn if they collaborated in the same article. We used the dataset from Ref[1] which spans a period between 1893 and 2006. To have the average degree in each instantaneous network as comparable as possible (see Fig. 3), we select a period of ten years, from January 1997 to December 2006. In this time window we register 96940 scholars who create 692667 connections. When we simulate SIR and SIS models on top of APS temporal network, we use periodic boundary conditions to let the disease dynamics evolve without late-time constrains.

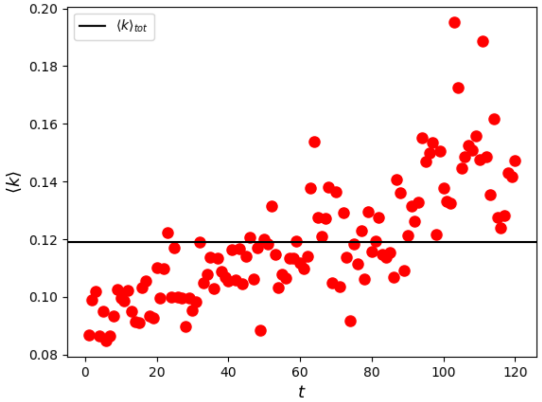

Figure 3. Average degree for each month in the selected subset of APS dataset (red circles), compared with the global average of all the ten years considered (solid black line). We label each month with increasing integer numbers from 1 to 120, where 1 represents the beginning of our sample, January 1997, and 120 the end, December 2006. We observe an increasing number of collaborations through years.

Using OSLOM, in Fig.4A we show that the integrated APS network is modular. Then, we apply the following degree-preserving randomization technique to destroy the network's community structure. We choose randomly a source node S_1 and, among its neighbors, we select randomly a target node T_1. We do the same for other two nodes S_2 and T_2. If the two pairs are equivalent or if a multi-edge will be created, we start back by selecting S_1 again and repeating the instructions. Otherwise, we swap T_1 and T_2 to have the new undirected links: $S_1 - T_2$ and $S_2 - T_1$. The edges are chosen within the same temporal network and the number of swaps is equal to the number of edges in that instantaneous network. So, the above procedure is applied for each instantaneous network. At the end, we integrate the randomized temporal network and use OSLOM to detect the community structure. In Fig.4B, we qualitatively prove that our degree preserving randomization destroys the network's community structure.
The options used in OSLOM are: -uw (to study undirected networks); -cp0.99 (to have communities as large as possible); -hr (to avoid to consider hierarchies); -r100 (to repeat 100 times the community detection). Since OSLOM finds communities in a non-deterministic way, last option is useful to get rid of stochastic fluctuations and have a more reliable community structure. Finally, we evaluate quantitatively the modularity of the two networks. For the original APS network, $Q = 0.6685$, and for its randomized counterpart $Q = 0.0937$.

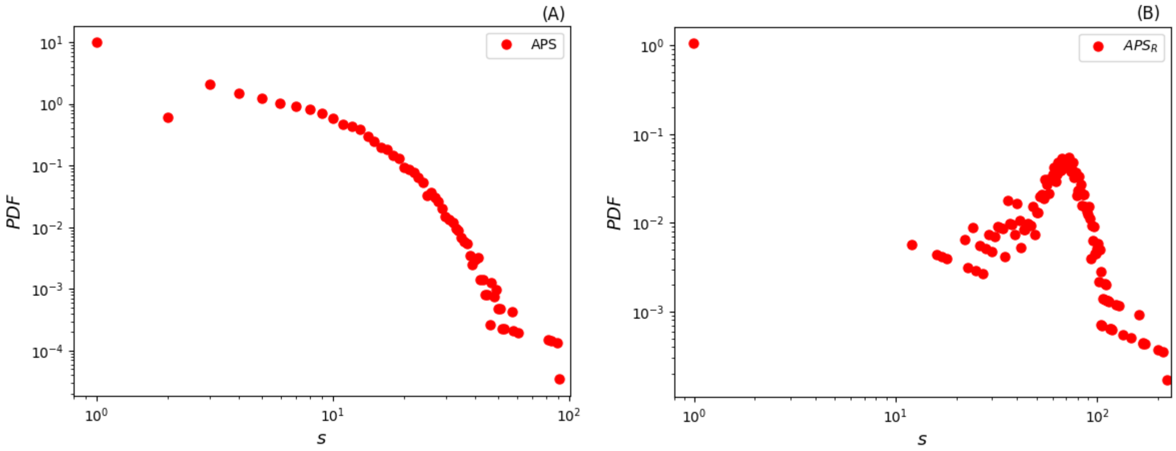

Figure 4. Panel A) Community sizes probability density function in the original network. Number of communities found is 10825, minimum community size is 1, maximum 91. Panel B) Community sizes probability density function after having applied the degree-preserving randomization. Number of communities found is 1489, minimum community size is 1, maximum 222. Note also that the shape of the distribution is completely different from panel A).

References

1. Radicchi, F., Fortunato, S., Markines, B. & Vespignani, A. Diffusion of scientific credits and the ranking of scientists. *Phys. Rev. E* **80**, 056103 (2009).
